# Effect of Milk Intake, Its Composition, and Fatty Acid Profile Distribution on Live Weight of Suckling Wallachian Lambs until Their Weaning

**DOI:** 10.3390/ani9100718

**Published:** 2019-09-24

**Authors:** Martin Ptáček, Michal Milerski, Luděk Stádník, Jaromír Ducháček, Vladimír Tančin, Jitka Schmidová, Michal Uhrinčať, Tereza Michlová, Lenka Nohejlová

**Affiliations:** 1 Department of Animal Science, Faculty of Agrobiology, Food and Natural Resources, Czech University of Life Sciences Prague, Kamýcká 129, 16500 Prague-Suchdol, Czech Republic; stadnik@af.czu.cz (L.S.); duchacek@af.czu.cz (J.D.); nohejlova@af.czu.cz (L.N.); 2 Genetics and Breeding of Farm Animals, Institute of Animal Science, Přátelství 815, 10401 Prague-Uhříněves, Czech Republic; milerski.michal@vuzv.cz (M.M.); schmidova.jitka@vuzv.cz (J.S.); 3 Research Institute for Animal Production Nitra, National Agricultural and Food Centre, Hlohovecká 2, 95141 Lužianky, Slovak Republic; tancin@vuzv.sk (V.T.); uhrincat@vuzv.sk (M.U.); 4 Department of Veterinary Science, Faculty of Agrobiology and Food Resources, Slovak Agricultural University, Tr. A. Hlinku 2, 94976 Nitra, Slovak Republic; 5 Department of Chemistry, Faculty of Agrobiology, Food and Natural Resources, Czech University of Life Sciences Prague, Kamýcká 129, 16500 Prague-Suchdol, Czech Republic; michlova@af.czu.cz

**Keywords:** genetic resource, ewe, growth intensity, Carpathian farming, fat, protein, lactose

## Abstract

**Simple Summary:**

Maternal milk represents a crucial source of nutrients provided to suckling lambs. We analyzed the properties of maternal milk relative to the live weight of suckling lambs, and confirmed positive impact of milk production, milk protein, and milk lactose on lambs growth intensity in general. Further analyses identified specific fatty acids contained in milk fat with positive effect to lambs live weight. Results highlighted important components of mother’s milk for optimizing lambs’ growth potential till their weaning.

**Abstract:**

This study determined effects of milk production, milk components, or fatty acids (FA) profile on live weight of suckling lambs till their weaning. Live weight (LW, kg) of 42 purebred Wallachian lambs (from 33 ewes) was recorded during four control days with approximately 30-day intervals during rearing. At the same time, their mothers were examined for milk production (kg), milk fat (g), proteins (g), lactose (g), and fatty acids (%) contents. Results investigated using linear regression analysis showed 5.93 kg (*p* < 0.05) increase of lambs LW corresponded with 1 kg of ewe’s daily milk production increase during the observed period. Similarly, significant 0.13 kg or 0.11 kg increases of lambs live weight corresponded with 1 g increase of milk protein or milk lactose at this time. Milk with higher prevalence of trans-palmitoleic acid, trans-vaccenic acid, cis-vaccenic acid, linolelaidic acid, linoleic acid, or conjugated linolenic acid (CLA) significantly improved lambs LW. Moreover, significantly positive Pearson partial correlation between LW and trans-vaccenic acid (r = 0.305) or CLA (r = 0.347) indicated on genetic correlation between these traits. Therefore, milk (natural or artificially supplied) with higher distribution of these specified FAs could improve lambs’ LW.

## 1. Introduction

Lambs growth intensity from birth until weaning is strongly influenced by a complex of characteristics related to the mother, called maternal abilities. Age, parity, ewes’ nutritional status, birth weight of lamb, lambing ease, or ewes’ behavior after parturition and during lambs rearing are some of the characteristics that influence a lamb’s growth, as [1,2,3,4,5,6] have demonstrated. Additionally, breeding values predicted for maternal effect help to increase lambs live weight by selection on maternal traits [7,8]. Another important factor reflected in maternal abilities is milk production and its quality, as it represents a crucial source of nutrients provided to suckling lambs. That is the reason for monitoring ewes milking ability [9] in non-dairy flocks, especially concerning low-input flocks or flocks under extensive management. Wallachian sheep are used in the maternal position while out-crossing as they have been historically adapted on breeding in very extensive conditions of Beskydy Mountains [10,11]. Their potential for milk production has been previously suggested by [12], demonstrating health beneficial fatty acid (FA) distribution in raw milk. However, in terms of mutton production the volume of milk produced, and its composition, as related to lambs live weight is of particular interest as well. Some previous studies confirmed positive effect of milk production [13,14] or milk lactose and protein distribution [15] on lambs growth intensity; however, this effect was not so obvious for milk fat [16]. Additionally, effect of particular FAs on lambs live weight should be informative. This was usually studied in the context of improving suckling lamb meat quality by polyunsaturated fatty acids (PUFA) enriched milk or milk replacers [17,18], or, recently, by direct diet fortification for specific oil supplements with high PUFA prevalence [19], demonstrating lambs’ growth and meat quality improvements.

The aim of this study was to determine the effects of milk production, namely, its milk components or FA profile on the live weight of suckling lambs until their weaning. Furthermore, to define those FAs in milk fat with significantly positive or significantly negative effects on lambs live weight.

## 2. Materials and Methods 

The study was performed from April to August 2015 on non-milked purebred Wallachian sheep, with their lambs managed under a traditional Carpathian farming system in the Beskydy Mountains (Moravian-Silesian region; 450–890 m above sea level altitude; local annual rainfall was 833 mm and the average annual temperature was 7.0 °C in the evaluated year). 42 lambs were randomly selected from the basic flock and their live weight until their rearing (LW; kg ± 100 g) was monitored on four control days: 27th April (the average 43rd day of age), 25th May (the average 71st day of age), 23rd June (the average 99th day of age), and 4th August (the average 142nd day of age). At the same time, their mothers (n = 33) were examined for milk production, and milk samples for solids and FAs estimation were collected. Milk production was measured in weight (MILK; kg). Milk fat, milk protein, and milk lactose were expressed as weight amount (g) contained in the milk. FA groups or particular FAs were expressed as percentage proportion (%) from FAs contained in the milk fat. This study was a part of the complex study bringing information about Wallachian sheep and showed their potential in current farming systems. Flock management, information about milk samples collection, and their analysis for solid components or for fatty acid profile analysis, as well as information about weighing lambs have been methodically described in [12,20].

Ethical approval was not required specifically for purpose of this study, as the investigations were carried out using data routinely collected throughout normal practice, in order to record performance. Additionally, procedures performed with the animals—for the whole project—were in accordance with Ethics Committee of Central Commission for Animal Welfare at the Ministry of Agriculture of the Czech Republic (Prague, Czech Republic), and carried out in accordance with Directive 2010/63/EU for animal experiments and Local Ethics Commission (No. 3/2015). Lambs’ live weight throughout the evaluation period was visualized by using a growth curve (interposed with polynomial function) from the base dataset MS Excel^®^ (MS Office, Microsoft Corporation, Redmond, Washington, USA).

All statistical analyses were performed using SAS 9.3. (Available online: https://support.sas.com/documentation/cdl/en/statug/63962/HTML/default/viewer.htm#titlepage.htm, accessed on 23 September 2019). [21]. Pearson partial correlation coefficients were used to express relations between residuals for LW on the one side and MILK, milk components, FA groups, and selected FAs on the other side. These relations were estimated after adjusting data for effects of control day, ewe age category, lambs litter size, lambs age, and for sex of lambs.

The second used approach were linear regressions of individual milk traits included into model equation for LW analysis of variance, which were estimated using generalized linear model (GLM) procedure of SAS software with significance level of *p* < 0.05, *p* < 0.01, or *p* < 0.001. Milk (kg), and milk components (g) obtained from twins were divided by 2 for this analysis. This data correction was performed based on assumption that the twins fed equally from their mother.

Y_ijklmn_ = µ + DAY_i_ + LS_j_ + AGE_k_ + SEX_l_ + age_m_ (DAY) + b *(MILK) or b *(PROT) or b *(LAKT) or b *(FAT) or b *(SFA) or b *(MUFA) or b *(PUFA) or b *(FAs: C_4:0_–CLA) + e_ijklmn_

where Y_ijklmn_ = lambs live weight, µ = mean value, DAY_i_ = fixed effect of the control day (four classes; i = first control day, i = second control day; i = third control day; i = fourh control day), LS_j_ = fixed effect of lambs litter size (j = singles; j = twins,), AGE_k_ = fixed effect of ewe age category (k = 1 and 2 years old ewes; k = 3 years old ewes; k = 4 years and older ewes), SEX_l_ = fixed effect of sex of lambs (l = males; l = females), age_m_ (DAY) = nested effect of age of lambs nested in control days of weighing, b *(MILK) = linear regression on milk production (144.72–2600 g), b *(PROT) = linear regression on milk protein content (9.49–114.14 g), b *(LAKT) = linear regression on milk lactose content (6.44–134.68 g), b *(FAT) = linear regression on milk fat content (15.51–148.98 g), b *(SFA) = linear regression on saturated fatty acid group content (45.18–75.53%), b *(MUFA) = linear regression on monounsaturated fatty acid group (18.90–46.23%), b *(PUFA) = linear regression on polyunsaturated fatty acid group (5.57–10.56%), b *(FAs: C_4:0_–CLA) = linear regression on fatty acid distribution (C_4:0_ = 0.94–2.50%; C_6:0_ = 0.83–2.41%; C8:0 = 0.64–2.51%; C_10:0_ = 1.49–9.06%; C_12:0_ = 1.15–7.94%; C_14:0_ = 3.75–16.74%; C_14:1_ = 0.02–0.56%; C_16:0_ = 16.68–27.87%; C_16:1T_ = 0.42–0.88%; C_16:1_ = 0.39–1.72%; C_17:0_ = 0.55–1.20%; C_17:1_ = 0.19–0.46%; C_18:0_ = 4.92–22.39%; ∑ C_18:1T_ = 0.52–15.23%; C_18:1n9c_ = 12.42–34.05%; ∑ C_18:1C_ = 1.16–2.54%; ∑ C_18:2T_ = 1.15–2.47%; C_18:2n6c_ = 1.03–3.67%; C_18:3n3_ = 0.69–2.92%; CLA = 0.13–4.41%), and e_ijklmn_ = residual error. 

## 3. Results and Discussion

**Figure 1:**
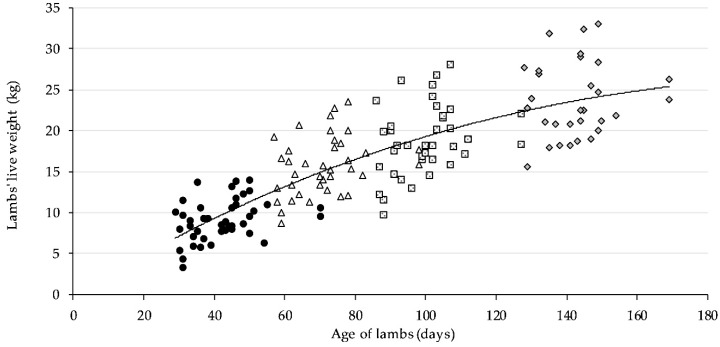
Growth curve (third order polynomial function) estimated for Wallachian lambs. 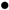 1st day of lambs live weight monitoring; 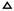 2nd day of lambs live weight monitoring; 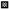 3rd day of lambs live weight monitoring; 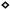 4th day of lambs live weight monitoring.

Information about milk production, milk composition, and FAs profile of Wallachian milk during lactation is presented in [12]. Figure 1 shows the dynamics of LW of Wallachian lambs throughout the observed period. 

Lambs LW was during this observation positively correlated with milk production (r = 0.257; *p* < 0.01), and all the evaluated milk solids (r = 0.248–0.305; *p* < 0.01) as documented in Table 1. This corresponded with previously published studies [9,22,23] that demonstrated a crucial effect of maternal milk on lamb growth rate. Additionally, Pearson partial correlation coefficients were used trying to suggest a genetic correlation between LW and milk production, or on LW and milk composition in case of a relatively small number of animals, as we had available. This supported positive genetic correlation between milk yield and average weight of the lambs weaned in the litter (r = 0.44) published by [24]; however, results for percentage milk components showed neutral (protein, r = 0.00; lactose, r = 0.01) or negative (fat, r = −0.31) correlation with average weight of the lambs weaned in the litter in their study. Table 1 shows more detailed view on milk fat illustrated by correlations analysis between LW and FA groups. FAs in saturated fatty acid (SFA) or monounsaturated fatty acid (MUFA) group indicated a neutral relation to lambs LW in general, while PUFA showed a significantly positive correlation (r = 0.194, *p* < 0.05). All these relations were further investigated using linear regression analysis showing a 5.93 kg (*p* < 0.05) increase of lambs’ live weight, corresponding with 1 kg of daily milk production increase during the observed period. Similarly, significantly positive linear regression was demonstrated for solids non-fat, when 0.13 kg or 0.11 kg increase of LW corresponded with 1 g increase of milk protein or milk lactose at this time. Importantly, both these linear regression were significantly positive; however, an error margin on the scales has to be considered when particular LW values are predicted using these regression coefficients. Contrary, a non-significant increase of LW was demonstrated by linear relation against milk fat content. Positive linear regression of milk production on lambs live weight was confirmed by a range of authors [13,14,15,16], when several of them also documented a significantly positive regression of milk protein or lactose. Milk fat was in a positive or in a negative linear relation with daily gains of twin lambs, as [16] have demonstrated; however, its r^2^ values were relatively low (ranging about 0.02–0.04) in experiments performed in their study. This supports only a minor linear effect of milk fat percentage that was observed in our results as well. However, a more detailed analysis of milk fat shows an evident significant increase of LW connected with increasing MUFA or PUFA. On the contrary, significantly negative linear regression was demonstrated for SFA. This indicates that generally respected FAs with beneficial health effect had positive impact on lambs’ growth abilities, while “health risk” FAs (lauric acid, C_12:0_; myristic acid, C_14:0_; palmitic acid, C_16:0_) contained in the SFA group showed a negative relation [25,26]. The effect of milk fat—as a whole—was eliminated as particular groups of FAs showed an opposed tendency. Therefore, more specific monitoring was aimed at identifying the effect of major FAs in milk fat on lambs live weight. 

Table 2 shows correlations of the major representatives of FA groups from mother’s milk to LW of lambs; three FAs showed a significant relation to LW. Stearic acid (C_18:0_), FA from SFA group, was negatively correlated to LW (r = −0.195, *p* < 0.05). Contrary, significantly positive correlation concerned trans-vaccenic acid (∑ C_18:1T_) (r = 0.305, *p* < 0.001), and conjugated linolenic acid (CLA) (r = 0.347, *p* < 0.001), representing FAs from MUFA and PUFA group, respectively. Prediction of lambs’ LW by changing course of FAs in milk fat was performed using linear regression analysis. FAs in SFA group showed neutral (butyric acid, C_4:0_; margaric acid, C_17:0_; stearic acid, C_18:0_) or significantly negative linear regression (caproic acid, C_6:0_; caprylic acid, C_8:0_; capric acid, C_10:0_; lauric acid, C_12:0;_ myristic acid, C_14:0_; palmitic acid, C_16:0_). Significantly positive linear-relationship concerned trans-palmitoleic acid (C_16:1T_), palmitoleic acid (C_16:1_), ∑ C_18:1T_, and cis-vaccenic acid (∑ C_18:1C_) (all MUFA groups) when 1% of C_16:1T_, C_16:1_, ∑ C_18:1T_ and ∑ C_18:1C_ corresponded with significant increases of LW 19.33 kg, 5.60 kg, 0.55 kg and 5.25 kg throughout the monitored time interval. Similarly, 1% of linolelaidic acid (∑ C_18:2T_) and CLA (all PUFA group) was reflected by a significantly increase of lambs live weight by 5.59 kg (∑ C_18:2T_) and 1.39 kg (CLA), respectively. In general only two FAs with significantly positive effect (both correlation and linear regression analyses) were representative of MUFA (C_18:1T_) and PUFA (CLA) group. Monitoring of FAs in milk fat is important for its health beneficial aspect in nutrition [27,28]. A strongly negative effect on health is particularly noted for lauric (C_12:0_), myristic (C_14:0_), and palmitic (C_16:0_) FAs [25,26]. Some positive occurrence of specific FAs from SFA group (such as C_6:0_ to C_10:0_) could be tolerated as they are of medical interest in humans: malabsorption syndromes, infant malnutrition, cardiovascular diseases, and nonallergenic properties [29]. However, none of these FAs showed some kind of positive relation to LW in our study. All named FAs were in significantly negative regression with LW. Contrary, FAs in PUFA and MUFA group are reported for their positive effect on cholesterol levels [29]. Additionally, CLA is frequently discussed in terms of anticarcinogenic effects [26] as well. Interestingly, generally respected positive effect of these FAs in metabolism should be indicated as an explanatory element of improved lambs growth demonstrated in the presented study.

## 4. Conclusions

This study described effect of maternal milk production and composition on lambs live weight. Milk production as well as milk protein and lactose contents showed a significantly positive linear relationship with lambs live weight. No such positive effect was obvious for milk fat percentage in general. However, the SFA group showed significantly negative linear regression, while a significantly positive relation was demonstrated for PUFA or MUFA. This positive effect of major FAs from PUFA or MUFA groups, especially concerning trans-palmitoleic acid, trans-vaccenic acid, cis-vaccenic acid (MUFA group), and linolelaidic acid, linoleic acid, or CLA (PUFA group). Moreover, significantly positive Pearson partial correlation between LW and trans-vaccenic acid or conjugated linolenic acid suggest a genetic correlation between these traits. Therefore, milk (natural or artificially supplied) with higher prevalence of these specified FAs could improve lambs’ live weight.

## Figures and Tables

**Table 1:** Regression-correlation analysis between Wallachian lambs’ live weight and milk production, milk compounds, or fatty acid groups during their rearing.

Linear Regression	*p*-Values for Fixed and Nested Factors in Model					Pearson Partial Correlations (r)
	DAY	AGE_ewe_	LS	SEX	AGE_lamb_ (DAY)	
LW = 22.22 + 5.93 × MILK *	***	n.s.	***	***	n.s.	r = 0.257 **
LW = 22.48 + 0.06 × FAT ^n.s.^	***	n.s.	***	***	n.s.	r = 0.248 **
LW = 21.73 + 0.13 × PROT **	***	n.s.	***	***	n.s.	r = 0.305 ***
LW = 22.40 + 0.11 × LACT *	***	n.s.	***	***	n.s	r = 0.261 **
LW = 52.01 − 0.45 × SFA ***	***	n.s.	**	***	n.s.	r = −0.078 ^n.s.^
LW = 13.20 + 1.37 × PUFA ***	***	n.s.	***	***	n.s.	r = 0.037 ^n.s.^
LW = 8.20 + 0.51 × MUFA **	***	n.s.	**	***	n.s.	r = 0.194 *

DAY = control days of lambs weighing; AGE_ewe_ = ewe age category; LS = litter size; AGE_lamb_ (DAY) = nested effect of age of lambs within control days weighing; LW = lambs live weight (kg); MILK = linear regression coefficient on milk production (kg); FAT = linear regression coefficient on fat content in milk (g); PROT = linear regression coefficient on protein content in milk (g); LACT = linear regression coefficient on lactose content in milk (g); SFA = linear regression coefficient on saturated fatty acid in milk fat (%); PUFA = linear regression coefficient on poly-unsaturated fatty acid in milk fat (%); MUFA = linear regression coefficient on mono-unsaturated fatty acid in milk fat (%); n.s. = non-significant; * = *p* < 0.05; ** = *p* < 0.01; *** = *p* < 0.001.

**Table 2:** Regression–correlation analysis s between lambs live weight and selected fatty acids in fat of Wallachian sheep milk.

Linear Regression	*p*-Values for Fixed and Nested Factors in Model					Linear Regression
	DAY	AGE_ewe_	DAY	SEX	DAY	
LW = 24.14 − 0.72 × C_4:0_^n.s.^	***	n.s.	***	***	n.s.	r = 0.086 ^n.s.^
LW = 28.53 − 4.25 × C_6:0_ *	***	n.s.	***	***	n.s.	r = 0.050 ^n.s.^
LW = 28.15 − 3.74 × C_8:0_ *	***	n.s.	**	***	n.s.	r = 0.048 ^n.s.^
LW = 29.05 − 1.32 × C_10:0_ **	***	n.s.	***	***	n.s.	r = 0.043 ^n.s.^
LW = 29.03 − 1.88 × C_12:0_ *	***	n.s.	***	***	n.s.	r = 0.061 ^n.s.^
LW = 32.72 − 0.88 × C_14:0_ *	***	n.s.	***	***	n.s.	r = 0.061 ^n.s.^
LW = 23.68 − 1.27 × C_14:1_^n.s.^	***	n.s.	***	***	n.s.	r = −0.005 ^n.s.^
LW = 42.12 − 0.80 × C_16:0_ *	***	n.s.	***	***	n.s.	r = 0.061 ^n.s.^
LW = 7.86 + 19.33 × C_16:1T_ ***	***	*	***	***	n.s.	r = 0.129 ^n.s.^
LW = 28.73 − 5.60 × C_16:1_ *	***	n.s.	***	***	n.s.	r = −0.130 ^n.s.^
LW = 16.30 + 7.81 × C_17:0_^n.s.^	***	n.s.	***	***	n.s.	r = −0.156 ^n.s.^
LW = 21.25 + 7.29 × C_17:1_^n.s.^	***	n.s.	**	***	n.s.	r = −0.104 ^n.s.^
LW = 24.94 – 0.10 × C_18:0_^n.s.^	***	n.s.	**	***	n.s.	r = −0.195 *
LW = 20.22 + 0.55 × ∑ C_18:1T_ *	***	n.s.	***	***	n.s.	r = 0.305 ***
LW = 18.46 + 0.26 × C_18:1n9c_^n.s.^	***	n.s.	***	***	n.s.	r = −0.128 ^n.s.^
LW = 14.24 + 5.25 × ∑ C_18:1C_ *	***	n.s.	***	***	n.s.	r = −0.051 ^n.s.^
LW = 14.52 + 5.59 × ∑ C_18:2T_ **	***	n.s.	***	***	n.s.	r = 0.033 ^n.s.^
LW = 17.89 + 2.81 × C_18:2n6c_^n.s.^	***	n.s.	***	***	n.s.	r = −0.096 ^n.s.^
LW = 19.95 + 2.31 × C_18:3n3_^n.s.^	***	n.s.	***	***	n.s.	r = −0.111 ^n.s.^
LW = 21.18 + 1.39 × CLA *	***	n.s.	**	***	n.s.	r = 0.347 ***

DAY = control days of lambs weighing; AGE_ewe_ = ewe age category; LS = litter size; AGE_lamb_ (DAY) = nested effect of age of lambs within control days weighing; C_4:0_–CLA = linear regression coefficients on lambs live weight by fatty acid (C_4:0_–CLA; %); LW = lambs live weight (kg); n.s. = non-significant; * = *p* < 0.05; ** = *p* < 0.01; *** = *p* < 0.001.
